# Christmas Day in a London Hospital

**Published:** 1887-01-08

**Authors:** 


### CHRISTMAS DAY IN A LONDON HOSPITAL.

By a Paying Patient.

A Dangerous Occupation.

Returning from the East about the end of 1877, suffering from reflex paralysis, resulting from a long and dangerous illness, I found, upon arriving in London, that a relative had arranged for my reception into one of the metropolitan hospitals, situate not nearly a hundred miles from Smithfield, that I might have the benefit of the eminent medical skill therein obtainable. On Christmas Eve the nurses were busily engaged in cleaning and decorating the ward, and I watched them with some trepidation, as they stood upon ladders, nailing up holly and other evergreens until a late hour in the night.

A Dark Cloud.

Christmas morning broke dull and lowering, but the sky brightened as the hours advanced, so that by eleven o'clock we looked forward to a cheerful day. About this time, however, one of the patients, who had been in an almost hopeless condition for a long period, gave evident signs of approaching dissolution, and before noon he had breathed his last. This event cast a gloom over the whole ward, and it was the opinion of the majority that the deceased had shown a want of consideration for his fellow-patients, by selecting Christmas Day, of all others, on which to "shuffle off this mortal coil." However, the nurses worked with a will: two stalwart porters brought a shell and removed the corpse; the atmosphere was perfumed with the smoke of burning cashmarilla chips, and by dinner-time we had somewhat recovered from the shock. Our fare consisted of the usual roast-beef and plum-pudding, to which I did scanty justice, my appetite at the time being by no means keen, and its edge had been effectually blunted by the scene I had so lately witnessed.

A Ray of Sunshine.

By tea-time, however, we were all feeling more cheerful; those who were considered to be well enough were told to get up and come to the table; but I wondered how we were to attire ourselves, as our clothes had been taken away, as usual, when entering the ward. This difficulty was solved in a manner novel to me. We were instructed how to envelope ourselves comfortably in our counterpanes, and until then I had no idea of the capacity of that useful covering for draping the human form, and I realised, for the first time, how the apparently impossible robes, in which holy men of old were, and are, invariably depicted, were practicable, although somewhat inconvenient. So striking was the resemblance, that when we were all enrobed, one of our number exclaimed, "I may not be the Apostle Paul, nor do I say I am;" but we all agreed that he much resembled that great missionary, as, with arm extended from his flowing robe, he addressed us.

Cake and Wine.

We were then regaled with plum cake, small cakes, grapes and oranges, and a glass of port wine apiece,—the last provided by our kind "Sister," and after sitting round a blazing fire and chatting for an hour or two the order was given to disrobe and return to our beds. By this time we were all feeling comparatively jolly, so conversation became almost general, and upon some one remarking that he had been for some years in the habit of rising at half-past four in the morning, the voice of a patient, who had not hitherto uttered an articulate sound, was heard for the first time. He who now spoke was one of the queerest-looking creatures I remember having seen, and my experience of the *genus homo* has been somewhat varied in Europe, Asia, and Africa. Imagine a man about sixty, with a long white face, elevated eyebrows, large drooping eyelids, a short nose, "tip tilted"; a long upper-lip, mouth drawn down at the corners, his head thickly covered with long, soft, red-brown hair, and, but for a fringe of the same under his chin, he would have presented a perfect picture of a querulous old woman. "Ugh!" grunted the incongruity, "how would you like to get up at 'arf-past two every morning for twenty years?" "May I ask," I inquired, "what avocation necessitated such exceptionally early rising?" "Creeses!" he replied. "Do you mean," I inquired, "water, ah! creeses?" "Yes! what other creeses should I mean?" "But," said, I persisting, "why should, ah! creeses more than any other esculent oblige you to turn out at such an unearthly hour?" "Well, you wouldn't get none if you weren't at Farrindon Market by three." "Ah!" said the patient in the bed next to mine, a lithographic printer, from Messrs. Waterlow's, who had been nearly dying from erysipelas, supervening upon an injury to his shin from one of the stones having fallen against it, "I believe there's a very large business done in creeses." "I should think there was," came the reply; "why,"—and here, becoming excited, he clutched the handle hanging from the top of his bedstead and struggled into an upright position—"why, there's one farmer down at Colchester who sends up as many as five ''underd' drapers a week to Farrin'don, large 'ampers, not little barskets." "There's more kinds of creeses than one," said the printer to me. "More than one kind! there's five or six kinds," said "Old Creeses," the name by which he went ever after. "I like the little brown ones," said the printer. "Little brown 'uns!" cried O. C.; "why, there's some of them little green 'uns, they eats like"—here his strength failed, and he fell back exhausted,—"they eats like sparrer-arrer-arrer-grass." From that moment until I left the hospital, about a week afterwards, "Old Creeses" never spoke again. Very shortly the night-nurse came on duty, the gas was lowered, silence was enjoined, and, after an hour or two of restlessness, dozing, I thought I was munching "creeses"; the brown ones were bitter, but the little green 'uns ate like "sparrer-arrer-arrer-grass." Then came "sweet oblivion," and so ended my Christmas Day in a London hospital.

